# A systematic review and meta-analysis on the prevalence and demographic risk factors of work-related musculoskeletal disorders in construction workers

**DOI:** 10.3389/fpubh.2025.1651921

**Published:** 2025-10-13

**Authors:** Weiner Santos, Alejandro Lorente, Carmen Rojas, Rui Isidoro, Ana Dias, Gonzalo Mariscal, Ahmed Hamdy Zabady, Rafael Lorente

**Affiliations:** ^1^International Doctoral School, University of Extremadura, Badajoz, Spain; ^2^Local Health Unit Litoral Alentejano (ULSLA), Santiago do Cacém, Portugal; ^3^Ankle and Foot Surgery Unit, Department of Traumatology and Orthopaedic Surgery, University Hospital Ramón y Cajal, Madrid, Spain; ^4^School of Industrial Engineering, University of Extremadura, Badajoz, Spain; ^5^University Polytechnic of Beja, Beja, Portugal; ^6^Life Quality Research Centre (LQRC-CIEQV), Santarém, Portugal; ^7^Institute for Research on Musculoskeletal Disorders, Catholic University of Valencia San Vicente Mártir, Valencia, Spain; ^8^Faculty of Science, Damanhour University, Damanhour, Egypt; ^9^Department of Orthopedic Surgery and Traumatology, University Hospital of Badajoz, Badajoz, Spain

**Keywords:** work-related musculoskeletal disorders, prevalence, construction workers, demographic risk factors, meta-analysis

## Abstract

**Introduction:**

Construction workers, who are constantly engaged in physically demanding tasks, face a significant prevalence of work-related musculoskeletal disorders (WMSDs). These conditions affect their quality of life and work performance and call for immediate attention. This study delves into the prevalence of WMSDs among construction workers and the associated demographic risk factors, highlighting the issue’s urgency.

**Methods:**

Our research process was thorough. Our search spanned electronic databases like PubMed, Scopus, Cochrane, Embase, and Web of Science. We included studies that involved adult construction workers reporting the prevalence of WMSDs such as back pain, neck pain, and other musculoskeletal diseases. The data were rigorously analyzed using R software, with subgroup and meta-regression analyses to assess the association between demographic factors and the prevalence of WMSDs.

**Results:**

The prevalence pooled by the meta-analysis was 59% for WMSDs from 14 studies with extensive study-level heterogeneity. Subgroup analysis illustrated differences by region, with higher prevalence in Asia (63%) compared to America (39%) and Africa (52%). Analysis of demographic factors identified the prevalence as significantly higher in the male gender (OR = 19.60). Workers over 40 were likelier to have WMSDs (OR = 39.04). Daily work hours were inconsistently associated. Lower back and shoulders were the most affected body regions.

**Conclusion:**

Our findings underscore the need for further research to identify other risk factors and design effective prevention strategies. The high incidence of WMSDs among construction workers, significantly related to demographic factors such as gender and age, calls for continuous investigation and the introduction of targeted interventions like work rotation, ergonomic training, and psychosocial support. These measures are crucial in preventing WMSDs and promoting the well-being and performance of construction workers.

**Systematic review registration:**

https://doi.org/10.17605/OSF.IO/BJ9KV.

## 1 Introduction

Construction workers are continuously exposed to strenuous physical activities, which leads to a significant prevalence of work-related musculoskeletal disorders (WMSDs) among employees (1). WMSDs resemble various injuries and illnesses that affect muscles, bones, tendons, ligaments, and nerves, causing pain and long-term disabilities in different body parts. These are highly detrimental to the employees’ quality of life and lead to premature retirement and decreased productivity, thus highlighting the need for intensive interventions. As a direct consequence of stressful activities associated with their job, construction workers are highly susceptible to the onset of WMSDs (2–4).

Approximately 50% of construction workers experience recurrent musculoskeletal pain throughout their working lives, with the incidence of the condition accelerating with the progression of time. The construction industry is famous for its numerous risky jobs involving heavy lifting, repetitive movements, awkward postures, and sustained physical strain (3, 5, 6).

Recent research indicates that construction workers face a heightened risk of musculoskeletal injuries compared to other occupational groups, underscoring the urgent necessity for targeted interventions and preventive strategies (7, 8).

Individuals in the construction industry encounter various risk factors, including persistent physical demands associated with transporting heavy materials and environmental issues such as inadequate training, substandard ergonomic procedures, and limited access to suitable tools. Furthermore, psychosocial factors in the workplace, such as stress and job dissatisfaction, can increase the incidence of MSDs among construction workers. Additionally, demographic risk factors contribute to the increasing prevalence of WMSDs, such as age, gender, years of experience, and work hours per day (5).

Laboratory tests and imaging modalities like X-rays are needed to diagnose WMSDs and exclude other causes. According to the treatment of these disorders, risk factors should be explored to personalize a supportive treatment plan. Physical and mental support is needed in such cases, so there is a continuous need to implement training programs focusing on ergonomics, stress management, and safe work practices to help minimize these hazards and build a safety culture in the construction sector. In addition to causing pain for the individual, MSDs also affect the sustainability of the workforce through increased absenteeism and reduced productivity, thereby affecting financial and workforce resources (5, 9–11).

WMSDs have also turned out to be a cost burden, with lost wages, medical expenses, and delayed projects since the condition is chronic and requires frequent treatment and physiotherapy. This necessitates the need for prevention (3, 12). Examples of ergonomic measures employed to minimize the frequency of accidents and ensure employee welfare include flexible equipment, job rotation, and psychological support mechanisms. The dual focus on prevention ensures longer-term running performance and safeguards the welfare of employees, thereby assisting the industry in coping with increased physical demands (11). The study aims to assess the prevalence of work-related musculoskeletal disorders and potential demographic risk factors.

## 2 Materials and methods

### 2.1 Study design

This study will employ a systematic review and meta-analysis approach to assess the prevalence of work-related musculoskeletal disorders and identify demographic risk factors across various occupational settings. This review adhered to PRISMA 2020 guidelines to ensure transparency, thoroughness, and reproducibility (13). The protocol was pre-registered in OSF through the following DOI: https://doi.org/10.17605/OSF.IO/BJ9KV.

### 2.2 Eligibility criteria

The eligibility of studies was defined using the PICO framework:

- Population (P):
- Adult construction workers aged ≥18 years, employed in any construction industry sector worldwide. Studies were eligible regardless of country, provided participants were identified as construction workers.
- Intervention/Exposure (I):
- Work-related physical demands and occupational tasks leading to musculoskeletal disorders.
- Comparator (C):
- Not applicable for prevalence studies. Where relevant, comparisons were made between subgroups (e.g., younger vs. older workers, males vs. females, different work-hour categories).
- Outcomes (O):
- Reported prevalence of WMSDs or musculoskeletal pain, with quantitative estimates. Studies that also reported demographic risk factors (age, gender, education, work experience, work hours) were considered.

*Inclusion criteria*: observational studies (cross-sectional, cohort, or case–control) reporting prevalence of WMSDs in construction workers; published January 2010–July 2025; English language; full text available.

*Exclusion criteria*: case reports, reviews, editorials, conference abstracts, and grey literature without primary data; studies without quantitative prevalence; studies on general laborers without specifically identifying construction workers; non-English publications without accessible translation.

### 2.3 Information sources and search strategy

A comprehensive literature search was conducted across several electronic databases, including PubMed (MEDLINE), the Cochrane Library, Scopus, Web of Science, and Embase, to capture relevant studies. The search was limited to studies published from January 2010 to 2025. The search strategy included a combination of keywords with Boolean operators as follows: ((“work-related musculoskeletal disorders” OR WRMDS) AND (prevalence OR “workplace injuries” OR “demographic risk factors” OR “musculoskeletal pain” OR “back pain” OR “neck pain” OR “carpal tunnel syndrome”)).

### 2.4 Study selection

Two independent authors (WS and GM) were assigned to screen the titles and abstracts of all studies identified in the literature search using Rayyan software. Full-text articles were screened for a more detailed evaluation (AL and CR). Any disagreements during this process were resolved by consulting a third reviewer.

### 2.5 Data extraction

Two independent authors (AZ, CR, AD, and RI) were assigned to extract data from a specific number of studies. Disagreements were resolved through consulting the senior author (RL). We used a standardized extraction form (study ID, year, country, design, sample size, demographics, WMSD prevalence overall/by body region, and available risk factors).

### 2.6 Quality assessment

The modified Newcastle–Ottawa Scale (NOS) was chosen for its validated use in cross-sectional prevalence studies, offering structured scoring across selection, comparability, and outcome domains. Studies will be rated as Good (7–9), Fair (4–6), or Poor (0–3).

### 2.7 Data synthesis and statistical analysis

We used R software 4.3.1 using the “meta,” “metafor,” and “dmetar” packages to analyze and create forest plots using different meta-functions. The odds ratios (OR) with a 95% confidence interval (CI) were calculated for dichotomous data. The prevalence of WMSDs was estimated using the meta proportion with the pooled prevalence of WMSDs from the included studies, with a 95% CI. A random-effects model was used to account for between-study heterogeneity. The *I*^2^ statistic was used to test heterogeneity. Subgroup analyses were also done by geographical region to determine the differences in WMSD prevalence between different environments. A meta-regression was also performed to assess the association between demographic risk factors, such as age, and the prevalence of WMSDs. Also, sensitivity analysis was performed to evaluate the strength of the results. This involved removing studies to assess whether their inclusion significantly impacted the findings. A significance level of *p* < 0.05 was adopted for all statistical tests as a cut-off point.

### 2.8 Publication bias

A funnel plot was employed to assess potential publication bias, and Egger’s test was used to statistically test for bias within the studies included. Asymmetry within the funnel plot may indicate that smaller studies with null results were less likely to be published and were thus underrepresented within the meta-analysis.

### 2.9 Ethical considerations

Ethical approval was not required since the current study is a systematic review and meta-analysis of published data.

## 3 Results

### 3.1 Study selection

**Figure 1:**
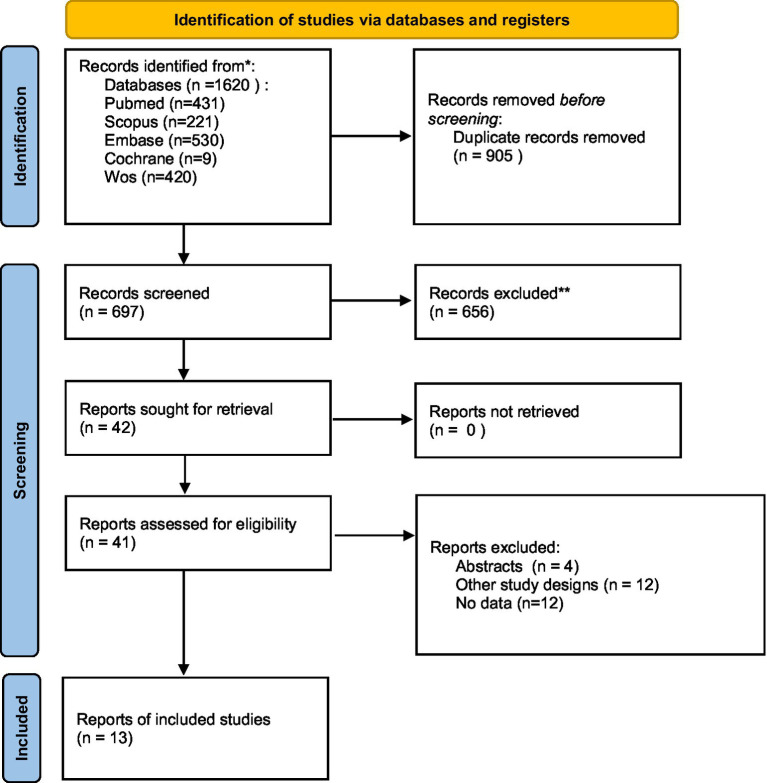
PRISMA flow diagram.

Our primary literature search, using our search strategy on different databases, yielded 1,620 citations. We found about 905 duplicates removed, 697 articles were screened in the titles and abstracts screening step, and we ended up with 42 articles to be screened in the full-text screening stage. Finally, 13 studies were included in our study. After reviewing the references in the included articles, we found that no additional studies met our inclusion criteria (Figure 1).

### 3.2 Study characteristics

Table 1 presents the main characteristics of the studies included. Thirteen studies included over 11 million workers, most of whom were men. These studies spanned Asia (India, UAE, Indonesia, Pakistan, Korea, China), Africa (Nigeria), and North America (United States), with the majority originating from Asia. The mean age ranged from 16 to 65 years, and the Body Mass Index (BMI) mean ranged from 20 to 35. Where reported, smoking prevalence varied markedly (≈2.7 to 69.6%), and educational attainment ranged from predominantly low in some cohorts (e.g., 84.2% low education in one Nigerian sample) to substantial college-level attainment in others (e.g., 40.1% in a U.S. study).

**Table 1:** Baseline characteristics of included studies.

Study ID	Country	Age means (SD)	Sex M/F	No. of smokers	BMI mean (SD)	Education
Reddy et al. (2016) (20)	India	NA	282/26	NA	NA	NA
Anwar et al. (2025) (4)	United Arab Emirates (UAE)	38.62 ± 9.06	All males	241 (69.6%)	27.68 ± 4.50	Lower than or equal to elementary school, Middle school, and Higher than or equal to the university
Kadir et al. (2025) (18)	Indonesia	NA	402/7	NA	NA	Low and High education
Ijaz et al. (2024) (14)	Pakistan	28.4381 ± 7.739	402/7	180 (30%)	28.4381 ± 7.739	11% with primary education
Jeong and Lee (2024) (17)	Korea	NA	156/22	NA	NA	NA
Lee et al. (2023) (3 23)	China	38.67 ± 9.05	337/43	NA	NA	Primary, middle, and high school
Kashif et al. (2022) (2)	Pakistan	34.49 ± 10.48	All males	249 (37.4%)	NA	NA
Dong et al. (2020) (15)	United States	NA	7275/719	NA	NA	40.1% colleague
Bashir et al. (2020) (8)	Pakistan	31.13	All males	NA	NA	NA
Wang et al. (2017) (6)	United States	NA	NA	NA	NA	NA
Egwuonwu et al. (2016) (19)	Nigeria	35.91 ± 8.46	All males	NA	26.16 ± 2.64	NA
Ekpenyong et al. (2015) (9)	Nigeria	26.42 ± 0.38	All males	32 (2.7%)	23.56 ± 0.26	84.2% low education and 15.8% high education
Bodhare et al. (2011) (16)	Karimnagar	16–64	197/32	NA	NA	Illiterate, primary, secondary, intermediate, and diploma education levels

### 3.3 Risk of bias assessment

Using the NOS tool, we identified three good-quality studies (2, 14, 15). Most of the studies were of fair quality (4, 6, 8, 9, 16–20), while one exhibited poor quality (3), as illustrated in Table 2.

**Table 2:** Risk of bias assessment using Newcastle Ottawa.

Study (Author, Year)	Selection (0–4)	Comparability (0–2)	Outcome (0–3)	Total NOS score (0–9)	Quality rating
Reddy et al., 2016 (20)	2	1	2	5	Fair
Anwar et al., 2025 (4)	2	1	2	5	Fair
Kadir et al., 2025 (18)	2	1	2	5	Fair
Ijaz et al., 2024 (14)	3	2	3	8	Good
Jeong and Lee, 2024 (17)	2	1	2	5	Fair
Lee et al., 2023 (3, 23)	1	0	2	3	Poor
Kashif et al., 2022 (2)	3	2	3	8	Good
Dong et al., 2020 (15)	3	2	3	8	Good
Bashir et al., 2020 (8)	2	1	2	5	Fair
Wang et al., 2017 (6)	2	1	2	5	Fair
Egwuonwu et al., 2016 (19)	2	1	2	5	Fair
Ekpenyong et al., 2015 (9)	2	1	2	5	Fair
Bodhare et al., 2011 (16)	2	1	2	5	Fair

### 3.4 Prevalence of WMSDs

**Figure 2:**
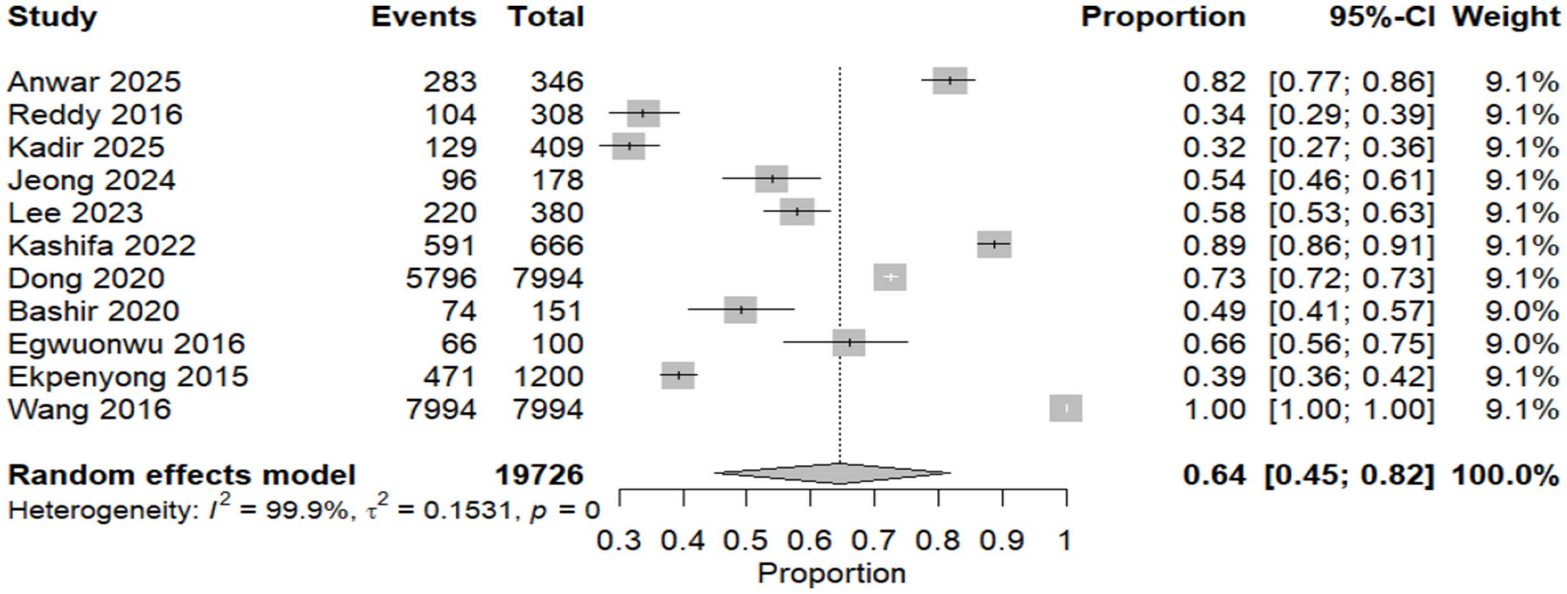
Forest plot of meta-analysis of the proportion of WMSDs among construction workers.

A meta-analysis of proportions was done to calculate the overall prevalence of WMSDs among construction workers. Fourteen studies were analyzed with a significant pooled prevalence of 0.64 (95% CI [0.45, 0.82], *p* < 0.0001). There was a high level of heterogeneity with an *I*^2^ value of 99.9%, indicating substantial variation in the effect sizes across studies, as shown in Figure 2.

Subgrouping analysis based on the country was done to reduce heterogeneity. The study was divided into three subgroups based on the country of origin: Asia, America, and Africa. For the Asian subset, the pooled prevalence was 0.63 (95% CI [0.45, 0.79]). The American subgroup yielded a lower prevalence of 0.39 (95% CI [0.03, 0.94]). The prevalence of the African subgroup was 0.52 (95% CI [0.00, 1.00]). The subgroup differences test revealed a nonsignificant (*p* = 0.0619) result, suggesting no differences in regional prevalence estimates (Supplementary Figure S1).

According to publication bias, a funnel plot was created with a weighted regression model with multiplicative dispersion. It showed no significant asymmetry, with a *t*-value of −1.61, 9 degrees of freedom, and a *p*-value of 0.1423. This indicates no evidence of publication bias. The limit estimate as the standard error approaches zero was 12.083, further supporting the lack of significant asymmetry (Supplementary Figure S2).

### 3.5 Prevalence among body parts

The pooled prevalence of WMSDs across different body regions showed significant variability, with heterogeneity (*I*^2^) values indicating substantial differences between studies. The Neck’s overall proportion is 0.23 (95% CI: 0.12–0.37) with a high *I*^2^ value of 99.6%. The overall proportion for the Shoulder region is 0.28 (95% CI: 0.15–0.44), with the *I*^2^ value being 98.5%. The overall prevalence for the Elbow is 0.13 (95% CI, 0.05–0.23), with the *I*^2^ value being 95.1%. The overall proportion for the Hip region is 0.16 (95% CI, 0.06–0.29), with the *I*^2^ value being 98.4%. The overall prevalence for the Knee region is 0.23 (95% CI, 0.11–0.37), with a relatively high *I*^2^ value of 98.8%. The overall prevalence for the Hand/Wrist is 0.17 (95% CI, 0.10–0.26), with the *I*^2^ value being 95.6%. The overall proportion for the Feet/Ankle region is 0.15 (95% CI, 0.06–0.27), with the *I*^2^ value being 98.7%. The overall prevalence for the Lower back region is the highest, with 0.32 (95% CI, 0.21–0.45), with the *I*^2^ value being 95.7%, indicating high heterogeneity in the estimates. The overall prevalence for the Upper back is 0.15 (95% CI, 0.07–0.25), with the *I*^2^ value being 96.3%, as indicated by Table 3.

**Table 3:** Results of meta-analyses of the prevalence of WMSDs among body regions.

Study	Pooled proportion	95% CI lower	95% CI upper	Heterogeneity *I*^2^
Neck	0.23	0.12	0.37	99.6
Shoulder	0.28	0.15	0.44	98.5
Elbow	0.13	0.05	0.23	95.1
Hip	0.16	0.06	0.29	98.4
Knee	0.23	0.11	0.37	98.8
Hand/wrist	0.17	0.10	0.26	95.6
Feet/ankle	0.15	0.06	0.27	98.7
Lower back	0.32	0.21	0.45	95.7
Upper back	0.15	0.07	0.25	96.3
Pain	0.79	0.42	0.99	97.6

### 3.6 WMSDs pain

A proportion meta-analysis was done to calculate the overall prevalence of musculoskeletal pain among construction workers. Four studies were combined with a significant pooled prevalence of 0.79 (95% CI 0.42, 0.99). There was also high heterogeneity with the *I*^2^ value of 97.6%, indicating a wide range of effect sizes across studies, as summarized in Table 3.

### 3.7 Comparing prevalence according to demographic data

#### 3.7.1 Gender

**Figure 3:**
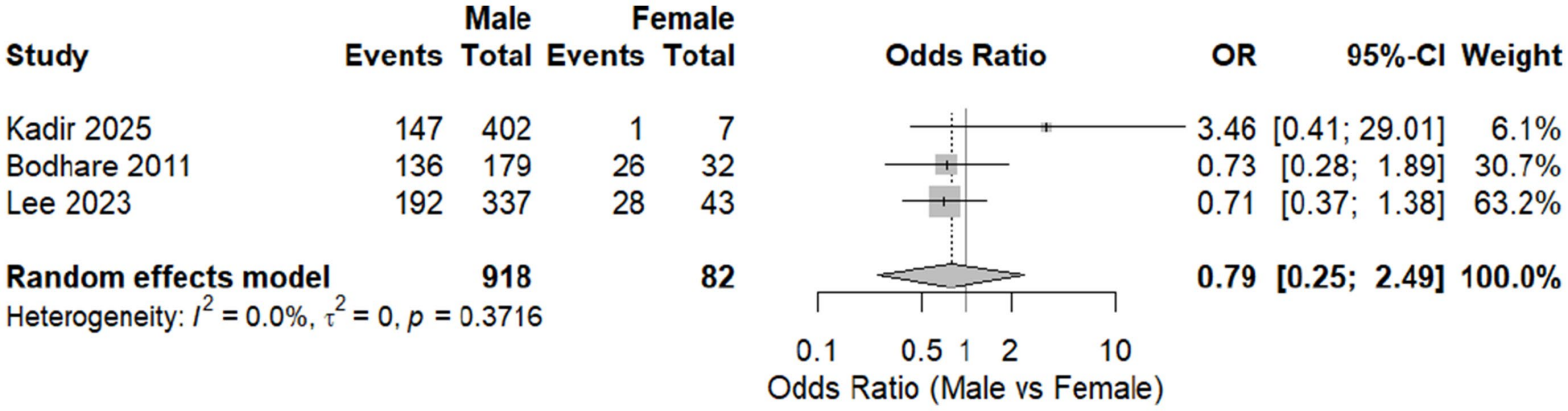
Forest plot of meta-analysis of the proportion of WMSD among males compared to females.

A meta-analysis comparing male to female prevalence was done, including studies that compared them. Nevertheless, males reported musculoskeletal disorders more frequently; there was no significant difference between males and females with an overall odds ratio (OR) across studies of 0.79 [95% CI: 0.25, 2.49, *p* = 0.4687]. There was no heterogeneity among included studies (*I*^2^ = 0%) (Figure 3).

#### 3.7.2 Age

**Figure 4:**
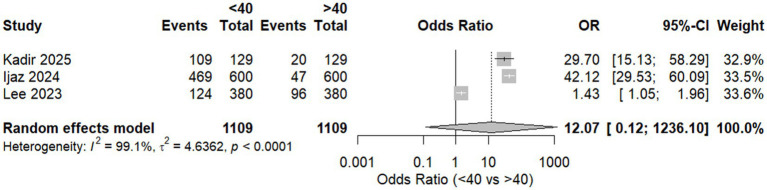
Forest plot of meta-analysis of the proportion of WMSD among workers aged <40 and >40.

Comparing workers aged less than 40 and more than 40, we found no significant difference between the two groups, with an overall OR of 12.07 [95% CI, 0.12, 1230.10, *p* = 0.1466]. There was a significant heterogeneity with *I*^2^ = 99.1%, as shown in Figure 4.

Sensitivity analysis was done by excluding Lee et al. (3); heterogeneity was totally resolved with *I*^2^ = 0%. Also, there was a significant shift in the context of the results, supporting that workers aged above 40 were more likely to develop musculoskeletal disorders with an OR equal to 39.04 and *p* = 0.0250 (Table 4).

**Table 4:** Sensitivity analysis results.

Group	Excluded study	Number of studies (*n*)	Heterogeneity (*I*^2^)	Overall proportion (OR)	95% CI
WMSD among workers aged <40 and >40.	Lee et al. 2023 (3)	2	0.0%	39.04	[6.26, 243.58]
Work hours and the prevalence of MSDs.	Lee et al. 2023 (23)	2	0.0%	18.35	[9.24, 36.46]

#### 3.7.3 Daily work hours

**Figure 5:**
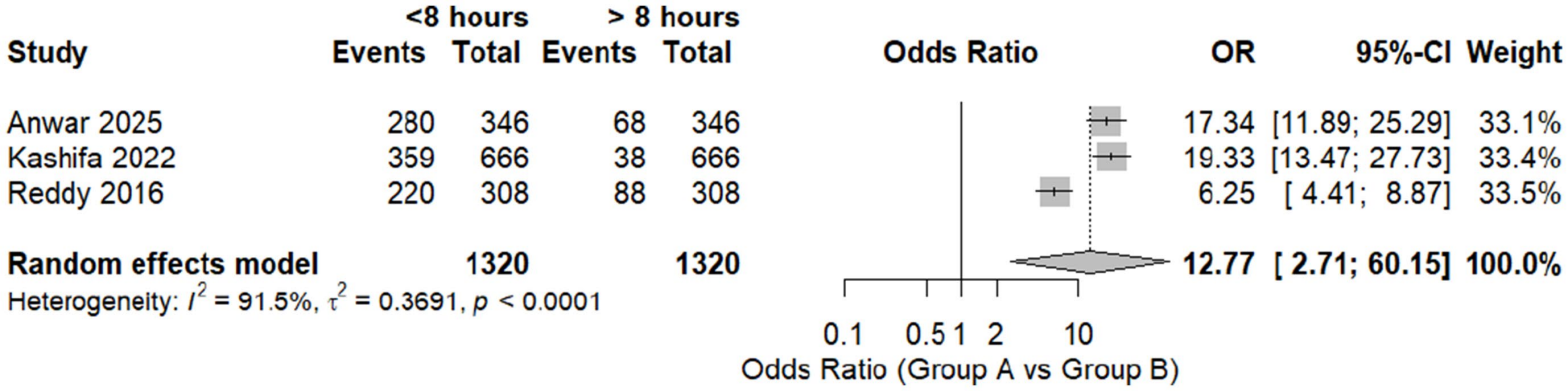
Forest plot of meta-analysis of the proportion of WMSD among workers who work for less than and more than 8 h daily.

Three studies were pooled to assess the relationship between work hours and the prevalence of MSDs. We found a significant difference between workers who work less than 7 h and those who work more than 8 h, with an OR of 12.77 [95% CI, 2.71; 60.1, *p* = 0.0194]. Significant heterogeneity was found with *I*^2^ = 91.1%, as shown in Figure 5.

A leave-one-out analysis was done, excluding Reddy et al. (20). We found that heterogeneity was resolved with no change in the context of the results with an OR of 18.3510 [95% CI, 9.24; 36.4637, *p* = 0.0118] (Table 4).

## 4 Discussion

Occupational disorders are increasing nowadays, with a focus on the construction sector; there is a horrible increase in musculoskeletal disorders due to the physical burden on workers (7, 21). This study focused on measuring the overall prevalence of MSDs among construction workers in different countries. Estimating prevalence can aid the community, and exploring the associated risk factors will benefit the community and workers by decreasing the economic burden and improving their health status.

We found that there has been an increasing prevalence throughout the past decade. In addition, demographic risk factors significantly contributed to this increase. Asian countries showed a more significant prevalence than African countries and America, with about 10 studies in Asian countries, only two in Africa, and two in America. Reporting to direct the world on updating and caring about occupational hazards is important. Further studies are needed to estimate such occupation-related disorders, which can lead to increasing morbidity and mortality ratios. Asian studies have revealed that while long work hours are prevalent, workers tend to adapt to the physical and mental demands through a combination of personal and institutional coping mechanisms (22).

There was a significant prevalence of MSDs among different body parts in construction workers; the lower back and shoulders revealed the highest reported prevalence, while the elbow was the least reported one, as explained in Table 3. Lee et al. and Egwuonwu et al. reported that the neck and lower back were the most frequent parts reported with musculoskeletal symptoms (3, 19).

Numerous risk factors for increasing musculoskeletal disorders include physical risk factors such as heavy lifting, repetitive motions, and awkward postures, which can significantly strain muscles, joints, and ligaments. Moreover, environmental factors include workplace settings, poor lighting, inadequate ergonomic design, or excessive noise. Exposure to extreme temperatures or vibration from machinery can further aggravate muscle and joint conditions, increasing the likelihood of injury. Importantly, demographic factors contribute to the increased risk of developing MSDs. Age is the most significant factor associated with different disorders, not only occupational-related disorders, due to a natural decline in muscle mass, bone density, and joint flexibility, making the old population more vulnerable to injuries (3, 6, 7, 21).

Additionally, individuals with a family history of musculoskeletal issues may have a predisposition to develop similar conditions. This study assessed the prevalence difference based on age. Although meta-regression of the prevalence and mean ages showed no significant relation between them, we found a significant increase in the prevalence among workers aged above 40 years old. This is consistent with previous studies. Oakman et al. found that Physical hazards and MSD risk vary by age group, with younger workers unaffected by physical hazards (12). However, older workers showed increased risk due to repetitive movements and awkward postures. Lee et al., also revealed a significant effect of age on the prevalence of MSDs (3). In contrast, Egwuonwu et al. showed no significant difference between age and WMSDs among construction workers (19). This controversy should be explored to understand the relation in further global studies comprehensively.

Gender also influences risk; although females are more prone to getting tired, there is an increasing trend in males. We found that males were more prone to developing musculoskeletal disorders. Still, a limitation can affect the results, as there was a significant difference between males and females working in the construction sector in the included studies. Lee et al. revealed similar results (23). It is important to note that while the male workforce in construction is traditionally more prevalent, this demographic shift might influence future trends in occupational health. As the construction industry becomes more inclusive, gender-specific interventions will be required to address the varying health risks for both men and women.

Interestingly, this study showed no significant difference between workers working for more or fewer than 7 h daily. Kashif et al. also reported no difference in musculoskeletal pain prevalence concerning daily work hours. Also, Lee et al. reported similar results equal to 0.237 (3). This finding challenges the assumption that prolonged daily working hours are directly associated with an increased risk of fatigue, stress, or musculoskeletal disorders. While the relationship between work hours and health outcomes has been extensively studied, the results of this study suggest that other factors may play a more dominant role in determining occupational health risks. Many studies have indicated that working long hours can lead to physical and mental health problems (2–4). This debate should be studied for a thorough understanding.

Also, our results revealed that individuals with more than 5 years of experience exhibited a lower prevalence, which suggests that experience helps workers to get familiar with the best ergonomic postures for their comfort. Anwar et al. reported that years of experience were significantly correlated with musculoskeletal problems in the neck only. Gajbhiye et al. found that years of experience significantly affected MSDs in the neck, shoulder, hands, wrists, back, legs, knee, and ankles (4, 5).

### 4.1 Limitations

Although this study performed a rigorous analysis to explore demographic risk factors and the prevalence of MSDs, several limitations should be acknowledged. Firstly, there is substantial heterogeneity among the included studies, with *I*^2^ more than 90% in most analyses. Although we conducted sensitivity analyses, meta-regression, and subgroup analyses to probe potential sources, heterogeneity largely persisted, and pooled estimates should be interpreted cautiously.

Also, most included studies originated from Asia, with fewer from Africa and America, limiting claims of global prevalence and generalizability. This geographical imbalance may limit the generalizability of our findings to other regions, particularly in areas with different working conditions, industry regulations, and ergonomic standards. Future research should aim to include a more diverse representation of global construction workers to better assess regional differences in MSD prevalence.

The reliance on cross-sectional designs and self-reported questionnaires introduces recall/reporting bias and excludes causal inference. Furthermore, most of the included studies predominantly examined male workers, reflecting the male-dominated nature of the construction industry. While gender comparisons were conducted, the lower representation of female workers may limit the accuracy of gender-based results.

Due to limited data, we did not assess job satisfaction, stress, or workplace ergonomics; future reviews should integrate these to improve comprehensiveness.

Also, our study assessed work hours as a potential risk factor for MSDs but found no significant difference between workers working more or less than 7 h daily. Nonetheless, this analysis did not account for variations in workload intensity, task complexity, or adequate rest breaks due to the lack of data reporting. These factors could play a crucial role in MSD development and should be considered in future studies to provide a more comprehensive understanding of work-related risk factors. Moreover, there was a lack of data about potential demographic risk factors, such as body mass index, ethnicity, and physical activities.

Due to the nature of our research question, only cross-sectional studies using self-reported questionnaires were relied on to assess MSD prevalence, which might introduce the risk of recall bias. This study lacked the assessment of psychosocial stress, job satisfaction, workplace ergonomics, and lifestyle factors. Additionally, this research design limits the ability to establish causal relationships between risk factors and MSDs. So, we recommend longitudinal studies to identify how occupational exposures accumulate over time and contribute to MSD development.

## 5 Conclusion

This study analyzed the prevalence of musculoskeletal disorders among construction workers across various regions and socioeconomic strata. The findings indicate a considerable overall prevalence of musculoskeletal disorders, with notable regional and gender disparities, particularly in the lower back and shoulders. The study highlights significant risk factors such as physical demands and age; nevertheless, it does not provide a clear correlation between daily work hours and the prevalence of musculoskeletal disorders. Examining the prevalence of musculoskeletal disorders among construction workers globally, while considering psychosocial, psychological, and demographic factors, remains essential.

## Data Availability

The original contributions presented in the study are included in the article/Supplementary material, further inquiries can be directed to the corresponding authors.
