# Supplementary material for: A systematic review and meta-analysis on the prevalence and demographic risk factors of work-related musculoskeletal disorders in construction workers
Source: Front Public Health. 2025 Oct 13;13:1651921. doi: 10.3389/fpubh.2025.1651921 (PMC12554755; doi:10.3389/fpubh.2025.1651921)
Supplement: Supplementary file 1 [file Table_1.docx]

**Supplementary Table S1.** Prevalence of WMSD according to demographics.

| Forest Plot | n. of included studies | Overall Proportion [95%-CI] | I² (Heterogeneity) |
| --- | --- | --- | --- |
| Male | 6 | 0.50 [0.24, 0.77] | 99.20% |
| Female | 6 | 0.60 [0.16, 0.92] | 88.80% |
| Years of experience more than 5 years | 5 | 0.32 [0.06, 0.77] | 99.50% |
