# Supplementary material for: A systematic review and meta-analysis on the prevalence and demographic risk factors of work-related musculoskeletal disorders in construction workers
Source: Front Public Health. 2025 Oct 13;13:1651921. doi: 10.3389/fpubh.2025.1651921 (PMC12554755; doi:10.3389/fpubh.2025.1651921)
Supplement: Supplementary file 2 [file Data_Sheet_1.docx]

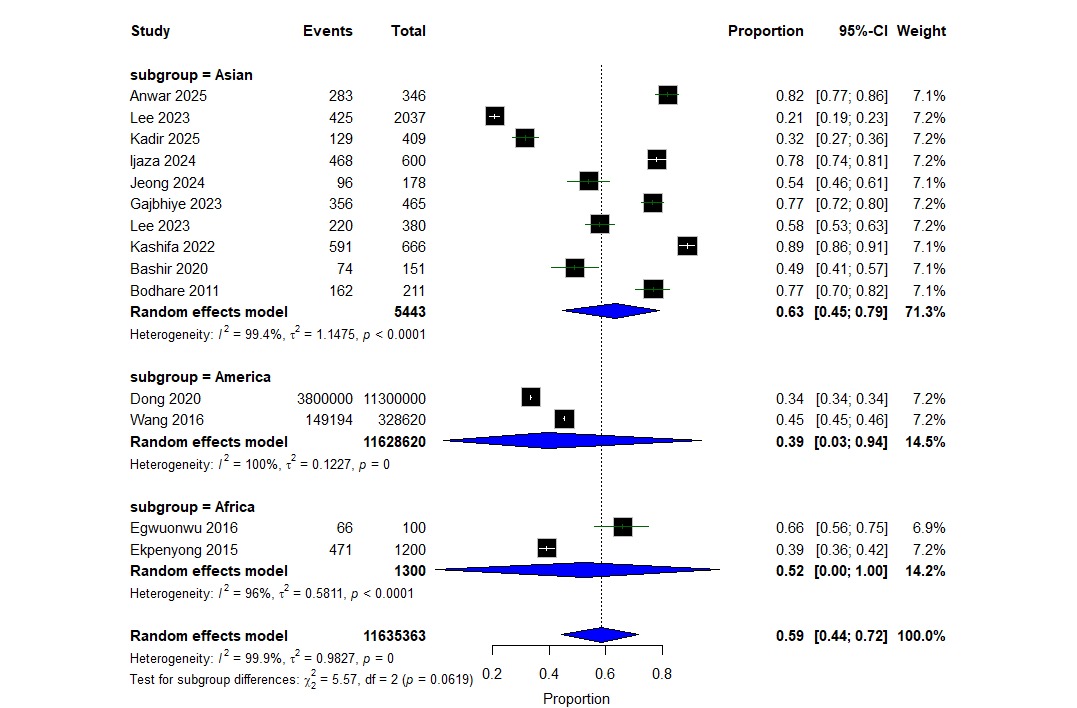


**Supplementary Figure S1**. Subgrouping analysis for the prevalence of WMSDs among construction workers.
