# Supplementary figures and images for: A systematic review and meta-analysis on the prevalence and demographic risk factors of work-related musculoskeletal disorders in construction workers
Source: Front Public Health. 2025 Oct 13;13:1651921. doi: 10.3389/fpubh.2025.1651921 (PMC12554755; doi:10.3389/fpubh.2025.1651921)

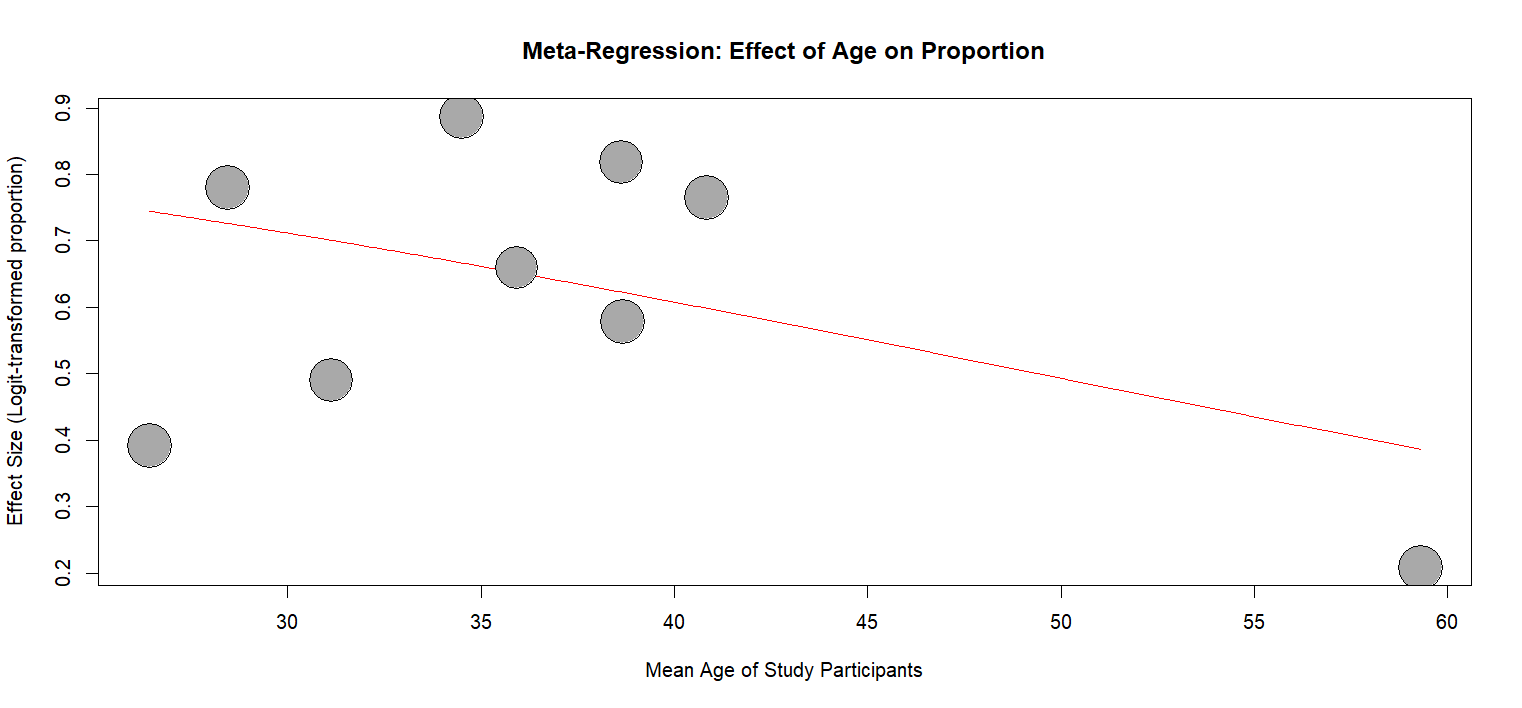


**Supplementary Figure S2.** Meta-regression of prevalence of WMSD based on age.

Supplement: Supplementary file 3 [file Data_Sheet_2.docx]
